# Does familial risk for alcohol use disorder predict alcohol hangover?

**DOI:** 10.1007/s00213-017-4585-x

**Published:** 2017-03-16

**Authors:** Richard Stephens, Kara Holloway, James A. Grange, Lauren Owen, Kate Jones, Darren Kruisselbrink

**Affiliations:** 1 Keele University, Keele, Staffordshire, ST5 5BG Newcastle, UK; 2 Psychology Department, University of Central Lancashire (UCLAN), Fylde Rd, PR1 2HE Preston, UK; 3 Health and Safety Laboratory, Buxton, UK; 4 Acadia University, Wolfville, Canada

**Keywords:** Alcohol hangover frequency, Alcohol hangover severity, Concurrent hangover, Alcohol use disorder (AUD), Family history

## Abstract

**Aims:**

Positive family history of alcohol use disorder (FHP), a variable associated with propensity for alcohol use disorder (AUD), has been linked with elevated hangover frequency and severity, after controlling for alcohol use. This implies that hangover experiences may be related to AUD. However, inadequate control of alcohol consumption levels, low alcohol dose and testing for hangover during the intoxication phase detract from these findings. Here, we present further data pertinent to understanding the relationship between family history and alcohol hangover.

**Methods:**

Study 1 compared past year hangover frequency in a survey of 24 FHP and 118 family history negative (FHN) individuals. Study 2 applied a quasi-experimental naturalistic approach assessing concurrent hangover severity in 17 FHP and 32 FHN individuals the morning after drinking alcohol. Both studies applied statistical control for alcohol consumption levels.

**Results:**

In Study 1, both FHP status and estimated blood alcohol concentration on the heaviest drinking evening of the past month predicted the frequency of hangover symptoms experienced over the previous 12 months. In Study 2, estimated blood alcohol concentration the previous evening predicted hangover severity but FHP status did not.

**Conclusions:**

FHP, indicating familial risk for AUD, was not associated with concurrent hangover severity but was associated with increased estimates of hangover frequency the previous year.

**Electronic supplementary material:**

The online version of this article (doi:10.1007/s00213-017-4585-x) contains supplementary material, which is available to authorized users.

Malaise experienced the morning after consuming alcohol, known as “hangover”, peaks as blood alcohol approaches zero (Stephens et al. 2008). Its biological mechanisms include reduced blood glucose, immune system imbalance and congener metabolism (Penning et al. 2010). Whether hangover influences alcohol use disorder (AUD) is unclear (Verster et al. 2010).

As family history of alcohol problems is associated with future difficulties with alcohol (Elliott et al. 2012) and AUD (Liu et al. 2004) research can assess factors to which family history positive individuals (FHPs) are differentially sensitive, and infer that these sensitivities are connected with AUD aetiology (Newlin and Thomson 1990). Knowing whether FHPs are more or less susceptible to hangover frequency or severity may help predict or alleviate future AUD. For example, the “traditional punishment model” (Span and Earlywine 1999) assumes that hangover is a natural curb on further alcohol consumption and predicts fewer hangovers in those prone to AUD, such as FHP individuals. Opposing this, the “withdrawal-relief model” (Span and Earlywine 1999) assumes that hangover is problematic because it invites further alcohol consumption to gain relief from its symptoms, and predicts more hangovers in individuals prone to AUD. Should either model be supported by evidence, this would inform researchers or practitioners aiming to reduce harms from AUD, possibly incorporating recall or experience of hangover or hangover-like symptoms and experiences as components within a methodological or therapeutic approach in this research.

A number of studies have found a positive association between hangover symptoms and family history of alcohol dependence, apparently supporting the withdrawal-relief model (Newlin and Pretorious 1990; Slutske et al. 2003; Piasecki et al. 2005; McCaul et al. 1991; Span and Earlywine 1999). However, a close analysis indicates a variety of methodological shortcomings.

Several surveys have assessed whether self-reported *hangover frequency over the past year* differs according to family history of alcohol problems (Newlin and Pretorious 1990; Slutske et al. 2003; Piasecki et al. 2005). These studies have consistently shown that FHPs have elevated hangover frequency, indicating that hangover experience may be predictive of AUD. However, while their findings are convergent, one limitation is that they may not have controlled adequately for the quantity of alcohol consumption. Two surveys (Newlin & Pretorious; Slutske et al.) that controlled for *typical* weekly alcohol consumption over the past year are problematic because similar weekly consumption levels could produce different hangover profiles if the entire amount was taken in one sitting or in lesser amounts more frequently (Verster et al. 2010). Thus, typical consumption may not control for a more hangover-prone drinking style. A composite measure (occasions in the last month of getting drunk, getting high and/or having five or more standard drinks) employed by Piasecki et al. is a more hangover-relevant measure of alcohol consumption. However, it may still fall short of characterizing hangover-prone drinking styles because of large individual differences in the quantity of alcohol consumed on one occasion to promote feelings of being drunk or high (Kerr et al. 2006) and because of the inter-individual differences in actual per-session consumption that this variable could include.

A credible alternative interpretation is that FHPs retrospectively report more frequent hangovers due to statistically uncontrolled heavier drinking. To address this, study 1 used an online questionnaire to assess whether hangover frequency in the past year would be elevated in FHPs after controlling for (i) estimated blood alcohol concentration (eBAC) arising from the *usual* amount of alcohol consumed in the past month, (ii) eBAC arising from the *highest volume* of alcohol consumed in the past month and (iii) drinking frequency in the past month. The period of 1 month was chosen for these estimates as it is long enough to be representative of participants’ general pattern of alcohol consumption but not so long that it would be susceptible to distortions due to memory decay. The eBAC calculation was based on participants’ sex, weight, height and the quantity and latency of alcohol consumed (Seidl et al. 2000).

Some further studies have assessed concurrent hangover in FHPs and FHNs (McCaul et al. 1991; Span and Earlywine 1999; Howland et al. 2008; Epler et al. 2014). The latter two found no difference in *hangover incidence* (i.e. the presence of a hangover compared with the absence of a hangover) despite relatively high quantities of alcohol consumed (0.10 g% BAC in Howland et al.; mean of 6.55 US standard drinks, equivalent to 92 g of ethanol in Epler et al.). In contrast, both McCaul et al. and Span and Earlywine found that *hangover severity* was greater in FHPs compared with FHNs following an alcohol challenge. However, in McCaul et al., the timing of assessments is a limitation. The dosage employed of 1 g/kg would be 70 g for a 70-kg adult. Assuming metabolism of 10 g alcohol per hour, it would take around 7 h for blood alcohol to reach zero. Yet the difference between FHP and FHN groups was most prominent at 3 h post-ingestion, diminishing thereafter, suggesting alcohol intoxication rather than hangover effects were detected. The conclusions of Span and Earlywine are questionable because a relatively low dose was applied (0.5 g/kg), likely to have resulted in a BAC of 0.05 g% estimated using the formulae provided by Seidl et al. (2000), assuming an average male of weight 70 kg, height 178 cm and a 1-h drinking period. This is considerably below 0.11 g%, the BAC threshold thought to produce hangover symptoms (Verster et al. 2010).

Summarizing, in research assessing concurrent hangover relative to family history of alcohol problems, studies assessing hangover *incidence* have found no relationship whereas studies assessing hangover *severity* appear to show a relationship but may be flawed for the reasons outlined. To address this, study 2 assessed concurrent hangover severity relative to FHP status in a naturalistic alcohol consumption paradigm taking into account alcohol consumption levels relative to body size.

## Method: study 1—online survey

### Participants

Two hundred ninety-nine individuals provided informed consent and clicked to start an online survey, but some completed only the first few items leaving 241 full datasets. As our research questions centred on hangover experience, self-declared non-drinkers were removed, leaving 198 cases. Individuals classified as neither FHN nor FHP were also removed, leaving 142 participants in the final analysis. These were adults aged 18–29 years (mean age 20.8 years, SD 2.3) including 90 females. The majority (*n* = 136) were UK citizens. The Keele University Research Ethics Panel approved the study.

### Materials

Hangover symptom frequency over the past year was assessed using the 13-item Hangover Symptom Scale (HSS; Slutske et al. 2003; Cronbach’s alpha = 0.84). The HSS was specifically designed to assess the frequency of hangover symptom occurrence 12 months retrospectively. Family history of alcohol problems was assessed using the 13-item versions of the F-SMAST and M-SMAST questionnaires (Crews and Sher 1992). Following Piasecki et al. (2005), participants were classified as FHN where *both* the F-SMAST and the M-SMAST scores were 1 or less; participants were classified as FHP where *either* the F-SMAST or the M-SMAST score was 5 or greater. Questionnaire items were used to record gender, age, academic performance (average module % last term) and age of first intoxication.

Self-reported height and weight measurements allowed blood alcohol concentration at the end of the drinking session (eBAC) to be estimated using the formulae provided by Seidl et al. (2000). The following drinking behaviours were recorded: the usual number of drinks consumed on days when they drank over the past month, the number of hours over which these drinks were consumed, the highest volume of drinks consumed on one occasion in the past month and the number of hours over which those drinks were consumed. This enabled calculation of two estimates of blood alcohol concentration—one based on the usual number of drinks per session reported for the previous month (eBAC-U) and one based on the highest volume of drinks per session reported for the previous month (eBAC-HV). Although eBAC-U and eBAC-HV highlight a participant’s pattern of drinking over the previous month, we assumed that this drinking pattern was indicative of the participant’s more general pattern of drinking.

### Design

A quasi-experimental cross-sectional design was applied comparing two independent groups. Based on F- and M-SMAST responses, an FHN group (*n* = 118) and an FHP group (*n* = 24) were identified. The dependent variable was hangover symptom frequency over the last 12 months assessed using the HSS. Statistical control was applied for estimated blood alcohol following the usual (eBAC-U) and highest volume (eBAC-HV) of drinks consumed in the past month as well as drinking frequency.

### Procedure

All students at Keele University were invited to take part in an online drinking survey in March 2014. The survey was hosted on *Google Drive©* and took approximately 20 min to complete. Identifying information was not routinely collected although 220 participants opted to provide an email address for entry into a draw with two £25 Amazon vouchers as prizes.

## Results: study 1—online survey

All variables followed a normal distribution although tending towards skew and platykurtosis in some cases. However, where appropriate transforms could be identified (e.g. logarithm; inverse), analyses yielded identical results and so only non-transformed analyses are reported. During the online data collection, 10 participants omitted to input their age and nine participants did not provide an answer to the item “age of first intoxication”. These participants were included in the analysis in order to maximize analytic power.

Descriptive data are shown in Table 1. The distribution of males and females was approximately the same in the FHN and FHP groups, chi-square = 0.010, df = 1, *p* = 0.922, phi = 0.020. Comparing the remaining variables in Table 1 across FHN and FHP individuals, only age *t*(130) = 2.653, *p* = 0.009, *d* = 0.465; age of first intoxication, *t*(131) = 2.097, *p* = 0.038, *d* = 0.366 and Hangover Symptom Scale score, t(140) = 3.601, *p* < 0.001, *d* = 0.610, differed significantly.

**Table 1:** Frequency of men and women and means (SDs) of age, BMI, academic performance, age first intoxicated, Hangover Symptom Scale (HSS) score and estimated blood alcohol concentration (eBAC) by family history of alcohol problems

Variables	FHN	FHP
	*n* = 118	*n* = 24
Sex		
Females	75 (64%)	15 (63%)
Males	43 (36%)	9 (37%)
Age*	20.58	22.00
	(2.04)	(3.18)
BMI	22.66	23.58
	(2.85)	(3.41)
Academic performance	64.49	62.50
	(8.46)	(11.52)
Age first intoxicated*	15.76	15.00
	(1.64)	(1.45)
HSS*	11.11	17.71
	(7.95)	(9.30)
Occasions of drinking in the last month	6.14 (5.04)	8.58 (7.93)
Units^a^ of alcohol consumed		
Usual drinks in previous month	4.97	4.50
	(3.00)	(3.05)
Most drinks in previous month	7.78	8.96
	(4.21)	(4.65)
eBAC (g%)		
Usual drinks in previous month	0.030	0.039
	(0.039)	(0.073)
Most drinks in previous month	0.066	0.094
	(0.068)	(0.108)

*FHN* negative family history, *FHP* positive family history

^a^A unit of alcohol is a UK standard measure containing 8 g of ethanol

*p < 0.05

### Is hangover greater in those with a positive family history of AUD?

A series of general linear model analyses were carried out using HSS score as the outcome variable. Three sets of these analyses were run—one set including FHP status and eBAC-U, a second set including FHP status and eBAC-HV and a third set including FHP status and drinking frequency. Initially, FHP status was entered as the sole predictor. Next, either eBAC-U, eBAC-HV or drinking frequency was entered as a second predictor, and the interaction term was inspected to check for regression slope homogeneity. Where the interaction was not significant, a final model was run omitting it.

The common first step for these analyses was a general linear model in which family history status (FHP v FHN) was included as the sole predictor of HSS score. This model indicated that FHP significantly predicted HSS score, *F*(1, 140) = 12.964, *p* < 0.001, *η* ^2^ = 0.085. Next, eBAC-U was added into the model and the interaction was inspected and found to be non-significant, *F*(1, 138) < 1.0; therefore, a final model was run including FHP and eBAC-U. In this model, FHP was a significant predictor of HSS score, *F*(1, 139) = 12.170, *p* = 0.001, *η* ^2^ = 0.081, and eBAC-U was a significant predictor of HSS score, *r* = 43.635, *F*(1, 139) = 9.087, *p* = 0.003, *η* ^2^ = 0.061. Adjusted *R* ^2^ for this model was 0.129.

A similar set of findings was found when eBAC-HV was included as a predictor in addition to FHP status. The interaction was not significant, *F*(1, 138) < 1.0, but in a model excluding the interaction, FHP was a significant predictor of HSS score, *F*(1, 139) = 10.292, *p* = 0.002, *η* ^2^ = 0.069 and eBAC-HV was a significant predictor of HSS score, *r* = 33.842, *F*(1, 139) = 15.261, *p* < 0.001, *η* ^2^ = 0.099. Adjusted *R* ^2^ for this model was 0.163.

In a model containing FHP, drinking frequency and the interaction of these two predictors the interaction effect was not significant, *F*(1, 138) < 1.0. Excluding the interaction, FHP was a significant predictor of HSS score, *F*(1, 139) = 11.649, *p* = 0.001, *η* ^2^ = 0.077 but drinking frequency did not predict HSS score, *F*(1, 139) < 1.0. Adjusted *R* ^2^ for this model was 0.076.

## Discussion: study 1—online survey

This online survey has replicated previously observed findings of increased retrospective ratings of hangover symptom frequency among FHPs controlling for alcohol consumption. Interestingly, when family history and blood alcohol were examined simultaneously, neither variable moderated or mediated the other, suggesting that positive family history of AUD and drinking behaviour explain separate aspects of retrospectively rated hangover frequency. Our study addresses some of the concerns raised in the introduction around the need to control for hangover-prone alcohol consumption rather than typical consumption. It is possible that the retrospective hangover frequency estimates provided by FHPs reflect a bias in past month drinking estimates; however, these data support the previous studies showing that FHPs report greater hangover frequency than FHNs over the past year, over and above alcohol consumption. This occurred despite more relevant measures of alcohol consumption—estimated blood alcohol following the largest amount consumed over the past month and drinking frequency—being taken into account.

However, while these data suggest possible predictive value of hangover in the aetiology of AUD, an alternative interpretation is that FHP individuals may be subject to a cognitive bias in recall accuracy such that they tend to recall more frequent hangovers than they actually experience. To elucidate this, study 2 assessed *concurrent* hangover severity in FHP and FHN individuals interviewed the morning after having consumed alcohol. Rather than using the HSS which captures the frequency of experiencing a variety of hangover symptoms over a period of time, study 2 used the Acute Hangover Scale (Rohsenow et al. 2007), an instrument designed to assess hangover severity by capturing the experience of a variety of hangover symptoms in situ. It was predicted that hangover severity would not be increased in FHP individuals compared with FHN individuals in line with the more robust previous studies assessing concurrent hangover in relation to family history.

## Method: study 2—quasi-experiment

### Participants

These were 49 adult drinkers aged 18–23 (mean 19.2 years, SD 1.20); 36 were female. Ineligibility criteria were recreational drug use in the last 28 days, medications causing drowsiness or affected by alcohol, diagnosed mental illness or alcohol/ drug problem, body mass index above 30, blood alcohol above zero and reporting zero regular weekly alcohol consumption. The Keele University Research Ethics Panel approved the study. Participants provided written informed consent.

### Materials

Concurrent hangover severity was assessed using the 9-item Acute Hangover Scale (AHS; Rohsenow et al. 2007; Cronbach’s alpha = 0.84). This scale was specifically designed for ongoing hangovers. Family history of alcohol problems was assessed using the 13-item versions of the F-SMAST and M-SMAST questionnaires (Crews and Sher 1992). The item “Did your father/ mother ever feel guilty about his/ her drinking?” was omitted in error; implications for scoring are discussed later. Tiredness (“at this moment”) was assessed using the Epworth Sleepiness Questionnaire (Johns 1991). Sex, age, ethnic origin, amount of sleep the previous night, number and type of alcoholic beverages consumed over the previous evening and the start and finish time of the drinking session(s) were self-reported. Height and weight were measured.

### Design

A quasi-experimental cross-sectional design was applied comparing two independent groups. Based on F- and M-SMAST responses, an FHN group (*n* = 32) and an FHP group (*n* = 17) were identified. Participants were classified FHN where *both* the F-SMAST and the M-SMAST scores were 1 or less; participants were classified FHP where *either* the F-SMAST or the M-SMAST score was 4 or greater. Participants were interviewed on a morning after drinking alcohol. The dependent variable was hangover severity assessed using the AHS. Statistical control was applied for estimated blood alcohol level the previous evening.

### Procedure

A screening interview ascertained inclusion criteria were met and usual drinking days of the week. Appointments made to attend the lab for assessment following usual drinking days could be cancelled where participants’ plans changed. Testing occurred from 9 am to 1 pm, lasting approximately 1.5 h. Blood alcohol levels were verified as zero using a Lion Laboratories Alcometer 500 electronic breath analyser. Some participants received course credit plus £10, while £20 was paid to those ineligible for course credit.

## Results: study 2—quasi-experiment

Most variables followed a normal distribution, the exceptions being usual weekly alcohol consumption level and estimated blood alcohol level, both of which evidenced positive skew and leptokurtosis. For both of these variables, a log transformation produced normality and the transformed variables were analysed. Descriptive data are shown in Table 2.

**Table 2:** Frequency of men and women, and means (SDs) of age, body mass index (BMI), hours of sleep the previous evening, Epworth Sleepiness Scale score, Alcohol Hangover Scale (AHS) score, units of alcohol consumed the evening before hangover, usual weekly number of units of alcohol and estimated blood alcohol concentration (eBAC) the evening before hangover by family history of alcohol problems

Variables	FHN	FHP
	*n* = 32	*n* = 17
Sex		
Females	23 (72%)	13 (76%)
Males	9 (28%)	4 (24%)
Age*	18.88	19.71
	(0.91)	(1.49)
BMI	23.16	22.33
	(2.64)	(2.93)
Hours of sleep previous evening	6.16	5.68
	(1. 82)	(1.14)
Epworth Sleepiness Scale score	7.00	8.18
	(3.41)	(3.64)
AHS score	27.81	28.12
	(10.18)	(8.11)
Units^a^ evening before hangover	10.61	11.47
	(6.53)	(7.23)
Usual weekly units	14.50	14.29
	(15.63)	(10.49)
eBAC (g%)^b^	0.12	0.20
	(0.08)	(0.24)

*FHN* negative family history, *FHP* positive family history

^a^A unit of alcohol is a UK standard measure containing 8 g of ethanol

^b^We chose not to exclude or modify two upper end outliers in the FHP eBAC data since there was no FHN v FHP difference on eBAC including these data

* *p* < 0.05

The distribution of males and females was approximately the same in the FHN and FHP groups, chi-square = 0.120, df = 1, *p* = 0.729, phi = 0.084. Of the variables listed in Table 2, only age differed across the FHN and FHP groups, *t*(47) = 2.430, *p* = 0.019, *d* = 0.709. Participants had consumed a mean of 87.3 g of alcohol, equivalent to around 1.2 g/kg.

### Is hangover severity greater in those with a positive family history of AUD?

A series of general linear model analyses were carried out using AHS score as the outcome variable. The first step was a general linear model in which family history status (FHP v FHN) was included as the sole predictor of AHS score but there was no effect, *F*(1, 47) < 1.0. Next, eBAC was added into the model and the interaction was inspected to check for regression slope homogeneity and found to be non-significant, *F*(1,45) < 1.0. In a final model omitting the interaction term, eBAC significantly predicted AHS score, *r* = 9.081, *F*(1,46) = 9.012, *p* = 0.004, *η* ^2^ = 0.164, but FHP did not predict AHS score, *F*(1, 46) < 1.0. Adjusted *R* ^2^ for this model was =0.128.

## Discussion: study 2—quasi-experiment

The prediction that hangover severity would not be increased in FHP individuals compared with FHN individuals was supported by the data. Contrastingly, estimated blood alcohol the evening before (eBAC), a variable capturing the quantity and duration of alcohol consumption relative to body mass, was a significant predictor of hangover severity. This replicates some previous findings (Chapman 1970; Huntley et al. 2015; Kruisselbrink et al. 2017), although peak blood alcohol level has sometimes been found not to predict hangover severity (e.g. Ylikahri et al. 1974), probably due to a stronger influence of individual differences in studies with smaller samples (Penning et al. 2010).

Experimenter error led to the omission of one item from the F- and M-SMAST questionnaires, necessitating lowering the criterion for FHP categorization from scores of 5 and above to 4 and above. While ensuring no potential FHPs were lost, others would have been included that would not otherwise have been. Some FHNs might also have not been so categorized had the missing item been present. Still, there is variation in the criteria for identifying FHN and FHP individuals such that FHPs have occasionally been designated with F or M-SMAST scores as low as 2 (Newlin and Pretorious 1990). Furthermore, a well-validated 9-item version of the F and M-SMAST questionnaires omits the same item (Crews and Sher 1992). We conclude that the omission has not significantly impacted the analyses.

## General discussion

Study 1 found greater self-reported hangover frequency the previous year in FHP individuals after controlling for hangover-prone drinking styles. Study 2 found that concurrent hangover severity was not raised in FHP individuals. One possible explanation for this discrepancy is that FHPs experience more variable symptom profiles from one hangover to another. If FHPs have a variable hangover symptom profile over time (that is, a more variable intra-personal hangover symptom profile), then this should manifest as greater variability in hangover symptoms within a group of FHPs at any given point in time. This would be due to some FHPs fluctuating towards reporting more severe symptoms, and others less severe at the time point in question. However, Table 2 indicates similar SDs for the variable AHS across the FHP and FHN groups. As a further check on this possibility, we assessed a tally of hangover symptoms reported via the AHS in study 2. We counted the number of symptoms from the AHS that were reported at a moderate or more severe level and observed that FHN and FHP individuals showed very similar tallies of symptoms (respective means 3.7, 3.9; respective SDs 2.2, 1.9; *t*[47] = 0.26, *p* = 0.796). Therefore, this explanation seems unlikely.

Taken together with prior research showing no increase in concurrent hangover incidence in FHPs, it appears that FHP individuals are not more susceptible to hangover severity or incidence per se, but that they evidence a retrospective bias in reporting increased hangover frequency over the previous year. One strength of the present research is that the questionnaires used to assess hangover frequency (study 1) and hangover severity (study 2) share a number of items including the three most commonly reported hangover symptoms: thirst, tiredness and headache (Rohsenow et al. 2007). This overlap of hangover assessment provides some confidence that a similar hangover construct was assessed across the two studies and consequently strengthens the case that the differing frames of reference (retrospective vs. immediate) underlie the findings. Nevertheless, future research could test the retrospective bias hypothesis more directly by asking drinkers to monitor drinking and hangover symptoms concurrently over a period of time, then retrospectively to report their experiences for the same period at a later time. If FHPs have a retrospective bias, they should show larger discrepancies between the self-monitoring and retrospective measures compared to FHNs.

That FHPs may have become sensitized to past hangover experiences is analogous to a variety of cognitive distortions observed among alcohol-dependent individuals. For example, Johnsen et al. (1994) found individuals with AUD showed slowed responses on alcohol-related words within a Stroop task. Our finding is novel because people at risk of AUD are thought to be insensitive to future adverse consequences relative to the short-term gains of intoxication (Cantrell et al. 2008); in other words, it is thought that for some drinkers the perceived positive benefits of consuming alcohol outweigh the influence of negative events. This is supported by data estimating that between 17% (Patrick and Maggs 2011) and 53% (Mallett et al. 2008) of college students rate hangover as not an adverse consequence of drinking.

Yet our data (study 1) suggest that people at familial risk for AUD are well aware of, and indeed may over-estimate, hangover frequency relative to drinking behaviour, compared with FHNs. It is not clear why this might be. One possible explanation of such a bias may be a veneration of hangover among individuals with a propensity for AUD as was suggested in a study of young Norwegian adults who talked about gathering the day after a party as a ritualistic and enjoyable way of coping with the discomfort of a hangover (Fjær 2012). However, guilt about drinking forms one of the 10 questions on the Alcohol Use Disorders Identification Test (AUDIT; Babor et al. (2001) and such guilt may encompass negative aspects of hangover, for example, letting down family members due to incapacitation the morning after drinking. Previously, it has been found that talking about hangovers in expressing intentions to avoid negative consequences of alcohol was marginally predictive of reduced alcohol consumption in college students (Mallett et al. 2015). Exploiting cognitive bias towards overestimating hangover frequency, perhaps foregrounding unambiguously negative aspects of hangover, may help practitioners support AUD-prone individuals in better managing their alcohol consumption. However, this recommendation is offered only tentatively pending further research assessing the extent to which negative aspects of hangover underlie such bias.

One limitation of both studies is that, while the analyses identified specific variables predicting hangover frequency and severity, the *R* ^2^ values associated with these models indicate that only a modest amount of variance in hangover, between 13 and 16%, was explained. It is known that factors beyond blood alcohol and family history of alcohol use disorder influence hangover outcomes, for example, guilt about drinking (Harburg et al. 1993). Further research should assess psychosocial influences on hangover alongside risk factors for AUD.

In addition, study 2 could be argued to be under-powered to detect differences in hangover severity across the FHP and FHN groups. However, the 17 FHPs in study 2 are comparable with the 16 and 20 FHPs in the two previous studies that purport to show a relationship between concurrent hangover severity and family history status (McCaul et al. 1991; Span and Earlywine 1999). Furthermore, the effect size of the FHP vs. FHN difference in hangover severity (AHS) score was *d* = 0.03, which is a very small effect size. Power analyses indicate that in excess of *N* = 34,000, participants would be required to detect an effect of this size, for alpha = 0.05 and power = 0.80. On this basis, a lack of statistical power seems an unlikely explanation for the null effects of study 2.

A further limitation is that the increased hangover frequency over the previous 12 months reported by FHP individuals in study 1 could be an artefact of inadequate control of alcohol consumption. However, given this is the fourth survey finding an increased frequency in retrospectively reported hangover among FHP individuals, and that study 1 controlled for hangover-prone drinking (quantity and frequency), this too seems unlikely. It is relevant that none of the retrospective estimates of alcohol consumption used in study 1 mediated the relationship between family history status and hangover frequency, but rather family history and alcohol consumption levels explained separate aspects of hangover frequency.

In conclusion, people at risk of developing AUD due to a positive family history do not appear to experience concurrent hangovers as any more severe than people with no family history yet FHPs tend retrospectively to overestimate hangover frequency. Further research should assess the extent to which negative aspects of hangover underlie this apparent cognitive bias. If negative aspects of hangover are important then practitioners may be able to exploit this to help AUD-prone individuals manage their alcohol consumption more effectively.

## Electronic supplementary material

ESM 1 (XLSX 39 kb).

ESM 2 (XLSX 35 kb).
